# Exploration of Social Media Usage Among Nurses in the European Region: A Cross-Sectional Study

**DOI:** 10.1097/CIN.0000000000001616

**Published:** 2026-08-20

**Authors:** Tiina Syyrilä, Laura Hako, Helena de Rezende, Ippolito Notarnicola, Marie-Louise Luiking, Jill Murphy, Helena Felizardo, Liz Westcott, Ilya Kagan

**Affiliations:** a Department of Nursing Science, University of Eastern Finland, Kuopio, Finland; b School of Nursing and Midwifery, University of Limerick, Limerick, Ireland; c Department of Medicine and Surgery, University of Enna “Kore”, Enna, Italy; d Leiden University Medical Centre, Leiden, The Netherlands; e School of Nursing and Midwifery, University of Limerick, Limeric, Ireland; f Scientific Pedagogical Unit for Fundamental Nursing and Basic Sciences, ESEUC – School of Nursing of the University of Coimbra, Coimbra, Portugal; g Faculty of Health, Science and Technology, Oxford Brookes University, Oxford, UK; h Nursing Department, Ashkelon Academic College, Ashkelon, Israel

**Keywords:** information dissemination, learning, nursing, social media, communication, Sigma Theta Tau International

## Abstract

The study aimed to explore nurses’ use of social media for networking, cooperation, and learning purposes. This cross-sectional study collected data from 158 nurses using an online questionnaire, primarily distributed through open social media for Sigma nurses across 19 European countries in 2023. Over half of the respondents were Sigma nursing members [n=105 (66.4%)]. PhD or a second academic degree was held by 58.2% (n=92) of the respondents. The questionnaire examined (1) background characteristics; (2) social media platforms’ use; (3) usage of social media for learning; and (4) networking. WhatsApp, Facebook, and Instagram were the most frequently used social media platforms. While most nurses did not perceive social media as a tool for learning, a significant portion reported using it for receiving, discussing, and posting knowledge, indicating a contradiction in perceptions of learning through social media. Social media was primarily used to stay updated on research, news, and webinars. Barriers to utilizing social media for learning addressed a lack of time, concerns over privacy, user skills, information overload, content reliability, and platform complexity. Social media can enhance learning and networking opportunities for nurses, but platforms must be selected and content tailored to meet their educational needs. Further research is necessary for generalizability, given the limited sample size and representativeness, and to optimize social media strategies for knowledge dissemination. This study adds to the literature by presenting indicative descriptive perspectives of predominantly highly educated nurses in the European region on social media use for professional networking, collaboration, and learning.

The rapid advancement of social media (SM) has profoundly transformed how individuals and professionals communicate, collaborate, and access information. The term SM was first introduced in 1994, and since then, the number of platforms and users has grown exponentially, establishing it as “one of the most important applications of the internet.”^1^ SM is described as a digital environment built by and for its users.^2^ It enables individuals to create public or semipublic profiles within a defined system and allows users to establish connections with others and view and navigate their connections.^3^ SM assists as a platform for collaboration and user-generated content^4^ and consists of applications that enable content diffusion, dialog, networking, and communication.^2^ It can be used for personal, professional, business, marketing, political, societal, and educational purposes, as well as for research, news updates, information exchange, and real-time connections.^2,5^ Various platforms, such as weblogs, Wikis, microblogging, and social networks, support these activities.^6,7^ In addition, scholars may use platforms like X (formerly Twitter), Facebook, Instagram, and in the region of China, mainly the WeChat application,^8^ for academic engagement and research dissemination.^6,9^ This study agrees on a social media definition verbalized by a systematic bibliometric analysis study covering peer-reviewed primary studies published over the last 2 decades; social media is “Internet-based channels that allow users to opportunistically interact and selectively self-present, either in real-time or asynchronously, with both broad and narrow audiences who derive value from user-generated content and the perception of interaction with others.”^6^ The authors also agree with Cathala et al^5^ interpretation of social media coverage, that messaging channels for a narrow, limited audience, such as WhatsApp, are considered social media.

In nursing, SM has emerged as a powerful tool for networking, professional development, and knowledge sharing.^10^ The literature shows little evidence of nurse scientists using SM to disseminate study results.^6^ However, students have indicated to be learning about research methods through social media.^10^ For instance, LinkedIn has enabled nurses to connect with peers, access up-to-date evidence, and participate in global conversations about nursing practice, education, and subjects related to the profession.^11,12^ The @WeNurses group on X, created to share knowledge and offer peer support, has amassed over 100,000 followers.^12^ SM connects nurses to professional development resources and informs them about the latest trends and practices. By following individuals, organizations, or groups, nurses can stay updated with current information and advancements in their field.^11^ Furthermore, the use of SM in nursing education has also grown significantly. Instant message platforms, accessible using smartphones, provide an inclusive and convenient way to support nursing education and peer communication.^5^ SM has also proven beneficial for nursing students in their academic and personal lives, helping them stay connected with university life and build a professional identity, share experiences, and even find job opportunities.^13,14^

In the light of O’Connor et al’s^6^ bibliographic analysis results, despite SM’s growing popularity, there is limited understanding of how nurses, compared to nursing students,^15^ across Europe utilize social media for professional purposes, particularly in the context of networking, collaboration, and continuous learning. Existing research has primarily focused on the use of social media in general or regarding maintaining professional boundaries and ethics,^15–18^ in specific regions or within isolated professional contexts,^19,20^ leaving a gap in our understanding of broader, cross-European trends. For instance, while studies have highlighted the benefits of social media for learning purposes^21^ or the role of microblogging and social networking-based communities (eg, Twitter) in fostering peer support,^12^ there is a lack of comprehensive data on how nurses across diverse European countries engage with social media to enhance their professional learning, practice, and networking. Furthermore, the potential of social media to support the strategic goals of nursing societies, such as Sigma Theta Tau International Honor Society of Nursing (Sigma Nursing),^22^ remains underexplored.

Sigma Theta Tau International Honor Society of Nursing (Sigma Nursing), founded in 1922, has over 100,000 nurse members in >100 countries globally and 12 regional chapters with around 500 members in Europe. The Sigma members are all registered nurses, graduated or at least studying at the master’s level in higher education, and they are, or aim to be, in a manager, leader, academic researcher, or educator position in nursing. Sigma organization intends to promote nursing scholarship, leadership, and collaboration by encouraging and supporting members through training, conferences, scholarships, international collaboration, and professional or academic possibilities, such as regional and international board memberships. Yet little is known about how to optimize social media to engage Sigma members and foster meaningful connections. Understanding social media use among nurses, such as preferred platforms, types of content consumed, and networking behaviors, can provide valuable insights for designing targeted strategies to enhance professional engagement and knowledge dissemination.

Several concerns and risks of SM in nursing have been discussed. Maintaining professionalism is paramount when using social media in nursing.^23,24^ Prior studies have recognized the importance of e-professionalism, meaning that nurses have appropriate professional boundaries in social media platforms,^24^ communication skills, and maintain privacy by managing relevant digital settings.^23^ The risks in using social media in nursing include, among others, using poor quality or misleading information,^10^ disseminating inappropriate pictures or using inappropriate vocabulary,^24^ failing to distinguish between private life and professional profile.^10^ A systematic review also shows that social media usage is not evenly distributed geographically^6^ or functionally the same everywhere.^10^ Considering the evidence above, this study aimed to explore how highly educated nurses in the European region use social media platforms for networking, collaboration, and learning.

## METHODS

### Study Design, Participants, and Setting

This descriptive, cross-sectional qualitative study collected data through an online questionnaire disseminated via Sigma Europe Region^25^ social media networks, as the research team consisted of Sigma Europe Region Communication Committee members. Recruitment occurred through the committee members’ professional networks and open social media channels, using convenience and snowball sampling.^26^ Because the invitation could be forwarded freely, the total reach was unknown; thus, a response rate could not be calculated.^26^ Thus, a prior power analysis result was not applicable. As access was not restricted to Sigma members, non-Sigma nurses also participated, which is reflected in the background characteristics. Accordingly, the study setting was the online environment, specifically the social media platforms and digital communication channels used for dissemination.

### Tools

Socio-demographic and professional characteristics were measured as follows: age, Sigma membership, nursing specialty and/or education level, gender, country, and language.

Social media use frequency, in general, regarding 10 social media platforms was measured using modified questions (from Part B) of a tool published by Rigamonti et al^27^ Rigamonti’s tool was based on the literature and expert opinions; validation was not described. The original tool’s developer was notified about the study via email, and the use was agreed upon by Rigamonti. The current questionnaire (Supplemental Text File 1, Supplemental Digital Content 1, https://links.lww.com/NXN/A0) consisted of the following sections: (1) socio-demographic and professional characteristics; (2) social media platforms’ use; (3) usage of social media for learning; and (4) networking.

The participants were asked to rank the degree of use of a defined social network on a scale between 1 (never) and 8 (almost every hour or more). For the analysis, the scale categories were combined into 5 categories: daily, weekly, monthly, yearly, and never.

In addition, participants were asked to name 2 popular social media platforms in their country in free text and rate the usage frequency of both according to a scale between 1 (never) and 8 (almost every hour or more).

Usage of social media for learning or networking was measured by the 7-item tool for each task (learning or networking). The items were constructed by the authors, based partly on the tools developed by Alharbi et al^13,28^ (from Part 2 questions) and Cathala et al^5^ (items concerning the use of SM). The corresponding and last authors of both studies were notified via email about the use of their tool in the study. The participants responded with a yes or no answer to the sentences representing the use of social media for learning or networking. Analyzing the scores will increase understanding of social media preferences for networking and learning purposes. Alharbi et al’s^13,28^ tool was based on literature, content validity validation (≥0.78), and pilot testing without a need for change. Cathala et al’s^5^ tool validation was not reported.

Qualitative information was collected using 2 open-ended questions: “What are the main barriers for you in using social networks for learning or networking purposes?” and “I would appreciate it if Sigma Europe offered other ways to share information: yes or no. If yes, please state what ways you would like to see for Learning or Networking.”

The content of the questionnaire was evaluated through discussion by an expert panel of 5 members from the Communication Committee of Sigma Europe Region^25^ members. Only the items with full consensus were included in the questionnaire.

### Procedure

The online version of the questionnaire was structured using the software program SurveyMonkey. The questionnaire language was English, which is the main communication language in the Sigma organization and between international members. However, the questionnaire was also translated into Portuguese and Italian by the authors, who were native Portuguese or Italian language users and used academic English regularly. Official back-translation was considered but was not conducted at the authors’ decision due to a lack of funding. Before conducting the final survey, a pilot survey was undertaken to ensure each translation was understood correctly. The distribution was carried out after receiving the approval of the Regional Committee of the Sigma Europe Region.^25^ An email explaining the research and providing links to all 3 language versions of the digital questionnaire (the respondents chose the most suitable for themselves) was sent to the national Sigma chapters to forward through member contacts. In addition, the questionnaire was distributed through the organization’s communication medium, the Sigma Europe Region website, LinkedIn, Facebook, X (Twitter was rebranded to X on July 23, 2023, and its domain name changed from twitter.com to x.com on May 17, 2024), and the Circle, which is an interactive social network for Sigma Nursing members.^22^ The survey was conducted between 26 June and 5 October 2023. The 3 reminder messages regarding the survey were distributed using the same method as the original invitation.

### Ethical Approval

Data were collected after receiving ethical approval from the Ethics Board of the Ashkelon Academic College, Israel (Approval number #52, December 5, 2022). The research was conducted in accordance with the Declaration of Helsinki of ethical principles for medical research involving human participants.^29^ Anonymity was guaranteed to all participants and secured by an online central collection of anonymous responses. At the beginning of the questionnaire, the participants received an explanation of their right not to participate in the study and the confidentiality of the responses. The last author saved the data in a protected OneDrive of the University. The researchers accessed the data through a password.

### Data Analysis

A priori power analysis was conducted using G*Power 3.1 for an independent-samples *t* test (two-tailed; effect size=0.40; α=0.05; power=0.90), indicating that 326 participants (164 per group) would be required. Because the achieved sample size and missing data prevented the planned comparative analyses, the calculated sample size target could not be applied in the final analyses. In addition, because recruitment was conducted via social media, the total number of individuals who received the invitation is unknown, and thus the response rate cannot be determined, nor is it required.^26^

Descriptive statistics [f (%)] were used to analyze data from socio-demographic and structured study variables, including Likert scale, dichotomic, and multiple-choice variables (Supplemental Text File 1, Supplemental Digital Content 1, https://links.lww.com/NXN/A0: the study tool). SPSS v.28 (IBM, US) was used to analyze descriptive data statistically. Qualitative free-text data from open-ended questions were manually analyzed in a Word table using the inductive content analysis method^30^ and quantification of sentences.^31^ The STROBE checklist was used to report the results (Supplemental Text File 2, Supplemental Digital Content 2, https://links.lww.com/NXN/A1).

### Handling Missing Data

To ensure the quality and reliability of the data set, a preliminary analysis was conducted to identify missing data. First, columns with missing values were removed, as they provided no relevant information for the analysis. This approach allowed for the preservation of data consistency and the creation of a more robust data set suitable for statistical analysis, reducing the risk of bias caused by incomplete data. Second, a tolerance threshold for missing data was applied to the rows: statistical units having >50% missing values were excluded. The response proportion of 70% to 85% of the survey questions per responder was preferred. However, the strict cutoff point would have lost valuable categorical and qualitative free-text data. Therefore, to maximize the use of qualitative data along with quantitative data, the cutoff point was set to 50% filled surveys by statistical unit. The high missingness concerns mostly individual respondent cases. The literature does not recommend imputation if the missing data is >40%.^32^ Altogether, n=91 statistical units were removed, and n=158 statistical units remained for the analysis. Proportions of missingness are reported for the analyzed variables.

## RESULTS

### Participants

The answers from 158 respondents showed that the mean age was 50 years, the mode age was 41 years (n=12), and the age range was 28 to 69 years (n=152; n=6 missing). Most of the respondents were female [n=116 (73.4%)]. Over half stated a nursing specialty or being an RN (registered nurse) with a PhD degree [n=53 (33.5%)] or having a second academic degree [n=39 (24.5%)] (missing, n=53). The respondents were most commonly from Portugal [n=37 (23.4%)], the UK [n=34 (21.5%)], or Ireland [n=22 (13.9%)], although 19 countries, predominantly from Europe, were represented (Armenia, Belgium, Canada, Croatia, Finland, France, Germany, Ghana, Ireland, Israel, Italy, Luxembourg, Netherlands, Norway, Portugal, Slovenia, Sweden, Switzerland, and United Kingdom). The most used languages on social media platforms were English [n=73 (46.2%)], Portuguese [n=33 (20.9%)], and Italian [n=9 (5.7%)], the 3 covering 72.8% of respondents. However, 16 different languages were named, of which 6 were mentioned only once. Over half of the respondents were Sigma nursing members [n=105 (66.4%)].

### Social Media Use by Nurses

As shown in Table 1, the leading social networks used daily by the respondents to this survey were WhatsApp, Facebook, and Instagram. Among the least useful are TikTok, Snapchat, and Reddit. These findings were also supported by qualitative and quantitative data, as shown in Table 2, with Facebook, WhatsApp, and Instagram being mentioned the most often in verbal reporting regarding the first and second most popular social media platforms. However, the results of the open-ended question also mentioned names of digital communication solutions, which were not traditionally classified as social media platforms (eg, email, SMS).

**Table 1:** Frequency of Using Social Media Platforms

Frequency	Daily, n (%)^a^	Weekly, n (%)^b^	Monthly, n (%)^c^	Yearly, n (%)^d^	Never, n (%)	Missing, n (%)	Total, n (%)
Facebook	81 (51.3)	16 (10.1)	14 (8.86)	11 (6.96)	35 (22.2)	1 (0.6)	158 (100)
LinkedIn	31 (19.6)	35 (22.2)	19 (12.0)	27 (17.1)	36 (22.8)	2 (1.3)	158 (100)
WhatsApp	145 (91.8)	4 (2.5)	—	2 (1.3)	5 (3.2)	2 (1.3)	158 (100)
Twitter	52 (32.9)	23 (14.6)	18 (11.4)	14 (8.9)	50 (31.6)	1 (0.6)	158 (100)
Instagram	63 (39.9)	19 (12.0)	9 (5.7)	13 (8.2)	52 (32.9)	2 (1.3)	158 (100)
TikTok	12 (7.6)	13 (8.2)	3 (1.9)	12 (7.6)	115 (72.8)	3 (1.9)	158 (100)
Snapchat	3 (1.9)	4 (2.5)	3 (1.9)	5 (3.2)	140 (88.6)	3 (1.9)	158 (100)
Reddit	3 (1.9)	2 (1.3)	—	2 (1.30)	148 (93.7)	3 (1.9)	158 (100)
YouTube	42 (26.6)	61 (38.6)	21 (13.3)	22 (13.9)	10 (6.3)	2 (1.3)	158 (100)
Pinterest	5 (3.2)	9 (5.7)	18 (11.4)	23 (14.6)	100 (63.3)	3 (1.9)	158 (100)

^a^ Almost every hour or more, at least once a day, many times per day, several times a day.

^b^ Once or twice a week.

^c^ Once or twice a month.

^d^ Several times a year.

**Table 2:** The First Popular Local Social Media Platform (Open-Ended Question)^a^

Local Social Media Platform^a^	n (%)
Facebook	19 (12.0)
WhatsApp	17 (10.8)
Instagram	7 (4.4)
Twitter	7 (4.4)
LinkedIn	4 (2.5)
Workvivo	3 (1.9)
Messenger	2 (1.3)
Signal	2 (1.3)
Threema	2 (1.3)
Yammer	2 (1.3)
Were mentioned just once^b^	19 (12.0)
None	12 (7.6)
I don’t know	6 (3.8)
Missing	56 (35.4)
Total	158 (100)

^a^ Free text answers.

^b^ BBC News, BeReal, Canvas, Dlrcoco, Email, “Facebook, Instagram, TikTok,” FNF Connect, Google, “Hospital/University networks,” Jodel, SMS, Spond, St Mellion hub, Telegram, Viber, Viva, Viva Engage, “WhatsApp, LinkedIn, Sms,” Zoom.

The second most popular social media platform among respondents was Facebook, with 14 mentions, accounting for 8.9% of the total responses. It is followed by the category “none,” which was chosen by 12 respondents (7.6%). Instagram ranks third, with 11 mentions (7.0%), while Twitter comes next, being mentioned 9 times (5.7%). Both LinkedIn and WhatsApp were cited 5 times (3.2%) (see Supplemental Table 1, Supplemental Digital Content 3, https://links.lww.com/NXN/A2 about the categorized results of “the second popular social media platform”).

A small number of respondents selected “I don’t know” (3 mentions, 1.9%), and Viber was mentioned 2 times (1.3%). Other social media platforms that were each mentioned once in the open-ended responses included Copains d’Avant, iMessage, Local IG Influencers, Meet, Quora, SharePoint, Sigma Chapter Facebook, Snapchat, TeamApp, Telegram, Threema, TikTok, Viva Engage, YouTube, and these together accounted for 15 responses (9.5%). In addition, there were 80 responses marked as “missing,” making up 50.6% of the total responses. Overall, the total number of respondents was 158. The actual survey population cannot be measured on open social media channels.

### Social Media Preferences Among Nurses for Learning

Most respondents did not perceive social media platforms for learning [n=115 (72.8%)]. Slightly under a third of respondents perceived using social media for learning and networking. However, based on the structured questions, over half of the respondents contradictorily answered using social media to receive, discuss, and post knowledge. The proportions of respondents using social media platforms are presented in Table 3.

**Table 3:** The Purpose of Social Media Platforms’ Use (n=158)

Purpose^a^	n (%)^a^	Missing^a^
For learning		
To receive knowledge contents	106 (67.1)	NA
To follow discussions about knowledge contents (passively)	88 (55.7)	NA
To post knowledge contents (passively)	79 (50.0)	22
I would appreciate it if Sigma Europe/my chapter used social media as a teaching aid or tool	76 (48.1)	29
To discuss study-related knowledge contents (actively)	61 (38.6)	NA
I do not use social media (or at least not at all for learning purposes)	24 (15.2)	28
For networking		
To receive knowledge contents	85 (53.8)	31
To follow discussions about knowledge contents (passively)	73 (46.2)	25
To post knowledge contents	66 (41.8)	25
I would appreciate it if Sigma Europe/my chapter used social media as a teaching aid or tool	64 (40.5)	42
To discuss study-related knowledge contents (actively)	57 (36.1)	39
I do not use social media (or at least not at all for learning purposes by contacting other members)	24 (15.2)	40

^a^ Agree + strongly agree.

On the basis of the open-ended question of how the respondents use social media specifically for learning [n=43 (27.2%)] (Supplemental Table 2, Supplemental Digital Content 4, https://links.lww.com/NXN/A3 concerning social media use for learning by respondents), they answered using it for: (1) to get information (n=8); (2) to communicate and consult (n=6); (3) to access education (n=10), webinars and journals (n=15); (4) to develop or get ideas (n=3); (5) to get updates (n=11); and (6) to network (n=3). Nurses, regarding the use of social media for learning, mostly (n=11) use it to get updates about news, research, or webinars/workshops [updates interesting news webinars/masterclasses (R39=respondent 39), updating on the news (R40), search for articles (R36)]. Under half of the respondents would appreciate it if chapters within the Sigma Europe Region would use social media as a teaching aid or tool [n=76 (48.1%)=agree + strongly agree]. However, slightly less was appreciated if Sigma offered other ways to share information [n=57 (36.1%); missing, n=34 (21.5%)].

On the basis of the open-ended question, the condensed purposes of other uses of social media (n=44 open-ended text responses) (Table 4) were as follows: to (1) access resources (n=10) (research papers, illustrations, topics or data for studies); (2) keep up to date nationally and internationally (n=1); (3) find conferences, events or jobs (n=6); (4) connect and consult (n=3); (5) to follow professionals or promote own profile (n=2); (6) to share research information (n=3); (7) to promote activities (n=2); (8) to find practical examples, expertise and innovations (n=5); and (9) to follow diverse profiles and journals (n=1).

**Table 4:** Additional Study-Related Purposes for Using Social Media

Purpose	Explanation of Use	Frequency of Responses Per Category
For accessing resources	R1 Conhecer recursos e inovação=knowing resources R2 Getting to know the studies that have been published R3 Explore research R4 Finding illustrations or citations (eg, Google Scholar) R5 Finding papers—summary of key findings R6 Finding resources R7 Research topics R8 I see social media as a potential data source for future studies. R9 Mainly, I use it to acquire information which I then can research further. Need to know more about it R33 Use Research Gate for example	10
Keeping up to date nationally and internationally	R10 Keeping up to date on what’s happening nationally and internationally	1
For conferences, events, jobs	R11 I use Twitter sometimes to read about research or conferences R12 Informed of available events/events and dissemination of scientific activities. Search for courses/training, scientific events that may be appropriate for my development/study R13 Searching for a job R14 To see study days of interest. R15 Search for training to complement my curricular enrichment. R16 Online seminars/lectures	6
For connecting and consultation	R17 Connect with authors networking and partnerships R18 Consultation, research R19 For meetings	3
Following professionals or promoting one's own profile	R20 I use social media to follow others professionally (passively) R21 I use social media to promote myself professionally (actively)	2
For sharing	R22 Sharing R23 Dissemination of published articles R24 Posting relevant news items, publications, alerts to grants, discussion topics (eg, the contributions nursing can make to the global climate changes)	3
Promoting activities	R25 Raising Sigma’s profile R26 Rather than loads of emails, try using social to bring people to that platform. Member rules to not overload people with information	2
For practical examples, expertise, and innovations	R27 Examples of practice or expertise Knowing innovations R28 To see what other hospitals are going do I can get ideas from them R29 To identify potentially new topics R30 Generating new ideas R31 Formation	5
To follow profiles and journals	R32 Following the profiles of the main journals, where recently published studies are published.	1
Other purposes	R34 YouTube R35 Zoom R36 I am retired so I am not active on social media R37 I don’t use it clearly for this purpose. R38 I don’t use social media (or at least I don’t use it at all for educational purposes) R39 no more reasons R40 Not at all R41 Don’t know R42 Not sure R43 Interaction with friends and family R44 Keep contact with friends and family (long distance)	11
Total		44

### Means for Enhancing Learning by Sigma Europe Region

Through analysis, social media as a means for enhancing learning by Sigma (n=33; missing, n=125) emerged as the overall theme with subthemes of: (1) dissemination of info about Sigma through websites, newsletters, and meetings; (2) disseminating articles, facilitating access to articles; (3) Ted Talks, Podcasts, Webinars, Workshops, digital learning and policy skills; and (4) using diverse platform channels (see Supplemental Table 3, Supplemental Digital Content 5, https://links.lww.com/NXN/A4 of categorized results about the “means for enhancing learning by Sigma”).

Respondents welcomed the opportunity to raise awareness, share knowledge internationally (R3), and disseminate information about Sigma. Suggestions included sharing through websites using the format of an annual meeting, a designated webpage with information links, or a monthly newsletter with updates on activities and links to further activities (R3, R8). Interestingly, an opportunity was noted for greater dissemination of the Sigma nursing platform in health infrastructures (R2).

Respondents suggested that learning could be enhanced through sharing and accessing dissemination of findings and relevant articles (R20, R1), with the provision of links to articles and research (R1), with 1 participant naming specifically “Research Gate” as a platform (R21) and another suggesting free access to international literature reviews and nursing studies (R2).

Ted Talk style of lectures (R24), podcasts (R19), webinars, and workshops appeared to be the modes identified by respondents to deliver webinars, continuous training, workshops, or short courses (R31, R7). One respondent called for ease of use and navigation of sites (R18). Digital learning, enhancing nursing skills, and how to change policy were suggested as learning content (R9, R12). Lunchtime availability was also highlighted (R17).

A call for use of diverse platform channels included Facebook, Instagram, LinkedIn, YouTube, Zoom for meetings, and WhatsApp (R28, R27, R11, R13, R30). There was an acknowledgment and sense of contentment with the current platform used by both the European Region and the individual’s local chapter (R16).

### Social Media Preferences of Nurses Networking With Others

The barriers to networking, based on free text responses (n=100; missing=58; total=158), concerned most often (1) a lack of time (19%) (see Supplemental Table 4, Supplemental Digital Content 6, https://links.lww.com/NXN/A5: barriers and challenges for networking by using social media). Lack of time constraints is a consistent barrier for many users, with responses highlighting difficulties in keeping up with platforms and balancing social media with work and personal life (R8, R59, R22, R40). Social media use is often seen as time-consuming (R31, R36, R79), and the complexity of platforms adds to the challenge (R38). For some individuals, too many existing obligations were felt as overwhelming, preventing them from engaging with social media (R56); (2) however, 10.1% did not recognize any barriers. A few respondents report no barriers to using social media for networking, as long as private messaging is included (R21, R23, R68); (3) lacking knowledge or fear of lacking skills for using SM were described as barriers by 6.3%. The respondents had a fear or lack of confidence in using social media (R11, R12, R77). This is compounded by a lack of knowledge or experience with technology (R32, R33, R35); (4) also, poor security and privacy (5.7%); and (5) getting too much information through too many channels (5.7%) were among the 5 most often described perceptions regarding barriers for SM use by nurses. The users worried about unsafe public sharing (R39, R52, R54) and the lack of data protection (R25, R53). Preferences for privacy-focused platforms such as Signal were mentioned (R53). The need for quality assurance in shared information is also noted (R55, R57). Some users prefer to focus on fewer platforms (R86) due to difficulty in navigating multiple social networks. With regard to too much information, too many channels to use and keep on, respondents express frustration with information overload, too many platforms, and frequent interruptions from ads (R15, R19, R51, R69, R82). There is also concern about missing important content due to the sheer volume of information (R59, R84).

In addition, the data showed that internet connectivity and accessibility issues, costs of data, and noninteractive formats are rarely perceived as barriers for SM use (R5, R29). Some respondents report difficulty accessing relevant information or navigating platforms due to complex formats or noninclusive designs (R7, R9, R18, R64). Accessibility needs to be user-friendly and nonintrusive for better engagement (R79).

With the lack of quality, reliability, credibility, and content, no specific responses were provided for this category. However, with opportunistic posting, unrelated or poor content, and oversharing of personal needs, wants, and opinions, marketing challenges identified include concerns over unreliable sources and low-quality content (R6, R13, R34, R65, R81). Some respondents express frustration with irrelevant content and personal oversharing (R78), as well as commercial interests disrupting group objectives (R62). Language barriers were rarely mentioned, particularly issues with using English or other unfamiliar languages (R10, R44, R58).

## DISCUSSION

This cross-sectional survey study, conducted via open social media, aimed to explore the social media platforms’ use by nurses and nurses’ usage of SM for networking, cooperation, and learning purposes in the European region, using a structured questionnaire with open-ended questions. Most respondents were women and had at least a second academic degree or a PhD. The results indicate that the views of nurses with higher education differ from those of nurses generally in clinical settings.^33,34^ The results represented at least 15 European countries and a variety of cultures and languages. The sample represented the average age of nurses within the nursing population, generally in Europe.^35^ The study produced novel insights into social media use among highly educated nurses, which seems to have been studied little, according to a 20-year bibliometric analysis conducted by O’Connor et al^6^ on the main research themes of SM in nursing and midwifery. RNs with a PhD may use SM in a different manner and for diverse purposes than nurses with European Qualifications Framework, EQF level 4, or a bachelor’s degree. Therefore, comparative studies between the educational level groups are needed in the future to facilitate tailored SM communication and education development for nurses at diverse educational levels. These groups were not compared in the current study due to the level of missing values.

Limited literature exists regarding the use of social media by graduated RNs.^18^ In the current study, the use of and activity in SM varied among the nurses, which is similar to the use of social media by nursing students.^36,37^ Consistent with previous literature,^38^ WhatsApp^39^ and Facebook^40^ were identified by the respondents as the most popular SM platforms, and nurses used them daily. In addition to WhatsApp and Facebook, nurses also used other SM platforms, such as Instagram and Twitter, daily.^39,40^

Nurses in the current study used SM to receive knowledge content, follow discussions about knowledge, access resources, keep up to date, and find events or jobs. In line with previous studies, SM was also used for educational purposes, including connecting and consulting others,^20^ in addition to knowledge sharing on research, and discussing study-related knowledge.^40,41^ Despite the factual use of SM by nurses in these activities, most nurses did not perceive that they were using SM for learning and networking. Interestingly, nurses were more passive recipients of knowledge and followers of discussions than active posters or participants in discussions. This highlights the potential to use SM for educational and knowledge dissemination purposes for nurses. However, it is crucial to understand the appropriate channels for sharing information and identify the main barriers to SM use for educational purposes.

The most frequently identified barriers to SM use among nurses were a lack of time and insufficient knowledge and skills to use SM. In a systematic mixed-studies review of 26 studies, similar skill-associated perceptions by nursing students were found.^36^ In the current study, perceived barriers to SM use included security and privacy uncertainty, as in previous studies,^12^ as well as the overwhelming amount of information received, and the use of too many SM platforms. Instead, in the Tan et al study,^36^ SM platforms used by student nurses were more numerous than found in the current study. Nursing students have previously preferred even more digital material than is available.^28^ Postings regarding research, scientific conferences, and practical knowledge seemed to attract the registered nurses and PhD-level nurses’ audience the most within this sample from Europe. While in a previous systematic review, nursing students often sought SM peer support for practical reasoning and information, or to develop their professional persona.^36^ The differences between the studies regarding the platforms used and the sought information by respondents may be explained by the educational background and different generations.

Other perceived barriers were connectivity, costs, accessibility, and interactivity of data, and the SM platform being complex to use and lacking user-friendly features. These findings are aligned with Tan et al's^36^ review on the influence of SM on student nurses. The perceived lack of quality and reliability of the content was hindering the use of SM by nurses. Inappropriate content^36^ and opportunistic posting were also identified as barriers to networking in SM platforms.

According to the identified barriers in this study and previous literature, the used SM platforms need to be easily accessible,^40^ and the information presented in a reliable and evidence-based form in SM platforms that are free to use. The information shared needs to be evidence-based in a form that can be easily read because of nurses’ reported time constraints. Thus, it is necessary to consider the Sigma Europe Region^25^ SM strategy regarding reaching the nurse audience by posting to the 4 most used SM platforms. The same information should preferably be published in a way that suits the characteristics of the SM platforms. It might also be useful to encourage nurses to discuss current issues in nursing on the Sigma Europe Region SM platforms to promote networking, share up-to-date information, and increase their own competence.^20,21^

Almost half of the nurses said they would appreciate it if the Sigma Europe Region or their own Sigma chapter used SM as a teaching aid or tool. However, it remained unclear how Sigma Europe Region SM platforms could further support this activity, as there was no space for free-text answers at this point. More research is needed to explore how educational support could be provided and which content is the most effective teaching aid or tool in SM.^7,21,41^ In addition, more information needs to be gathered from nurses to find out preferences, effectiveness, and usability of the information sharing and networking activities used by nurses in the European region.

The study has limitations that should be considered when interpreting the results. The results may reflect respondents’ general social media experiences rather than specifically focus on education and learning, though the professional scope was stated at the beginning of the questionnaire. Given the respondents’ mean age, the results might not necessarily reflect younger nurses’ preferences regarding the use of SM. The study participation link was shared in Sigma SM platforms; however, surprisingly, nearly half of the respondents were nonmembers. Furthermore, not all countries in Europe have a Sigma chapter, which resulted in the inability to report results covering the whole of Europe, though most of the countries are included. Furthermore, twelve respondents were living in non-European countries (eg, Armenia, Canada, Ghana, or Israel); these respondents are likely to be immigrants, exchange researchers, or support members of European Sigma chapters. Therefore, the results represent the views of half Sigma member nurses and half nonmembers, mainly in the European region. In addition, the respondents’ education level was higher than that of nurses working in the clinical field. Thus, the results might not represent the views of the average staff nurse; however, they bring novel, indicative information on the topic. There is a minor possibility that some respondents were not nurses due to the use of SM for data collection; however, this is unlikely. The free-text answer questions yielded a limited number of answers; thus, due to missing explanatory power,^27^ these should be considered rather as supporting original samples for the statistical descriptive results. The results are recommended to be interpreted as indicative and cautious, bearing in mind the proportions of missing data and the data collection method via an open social media platform. Because the current tool was just partly based on the validated tool and furthermore modified for the current study, the development of the tool with validity and reliability measurements in the future is recommended, as well as two-way translations. Another limitation concerns the reliability and validity of the instruments used. Although some items were adapted from previous literature, not all measures had undergone formal psychometric validation. Furthermore, internal consistency indices such as Cronbach’s α were not systematically assessed, which restricts the possibility of drawing firm conclusions on the robustness of the constructs. Finally, the high proportion of participants with doctoral degrees reduces the representativeness of the sample, limiting the generalizability of the findings to the broader nursing population.

## CONCLUSIONS

This study provides indicative insights into the use of social media for networking, microblogging, cooperation, and learning predominantly among highly educated nurses in the Sigma European Region. Despite the widespread use of social media platforms such as WhatsApp, Facebook, Instagram, and X (formerly Twitter), nurses who responded generally engaged more as passive consumers of information rather than as active contributors. These highlight the potential of social media as an educational tool. The perceived barriers that hinder full utilization of social media were time consumption, concerns over privacy, user skills, information overload, content reliability, and platform complexity. The results suggest that improvements in social media platform accessibility and user-friendly features are necessary to engage nurses in social media use for professional and academic purposes. Moreover, ensuring the delivery of evidence-based, easily digestible content is crucial to meeting the social media user needs of busy nurses.

## Supplementary Material
