# Supplementary material for: Exploration of Social Media Usage Among Nurses in the European Region: A Cross-Sectional Study
Source: Comput Inform Nurs. 2026 Aug 20;44(11):e01616. doi: 10.1097/CIN.0000000000001616 (PMC13727034; doi:10.1097/CIN.0000000000001616)
Supplement: Supplementary file 1 [file nxn-44-e01616-s001.pdf]

Supplemental Text file 1

SDC 1**:** The study tool

The research questionnaire on social media use

Greetings,

You are asked to participate in a study that examines the patterns of social media use for learning and collaboration purposes by the members of the Sigma Europe chapters. The questionnaire is anonymous. The data will be used for research purposes only. The details collected will remain confidential.

Thank you for your collaboration

Please mark the appropriate answer or fill in the blanks

Part A

1. What is your year of birth? ____________
2. Please enter the name of your Chapter: To add a list of chapters
3. Please enter your nursing specialty
    - Registered Nurse
    - Registered Nursing with first academic degree
    - Registered Nursing with second academic degree
    - Registered Nursing with PhD
4. Gender: a) male b) female c) other
5. Country: ________________________

Part B

Social Media Use* (such as liking, commenting, sending messages, and otherwise engaging with other users)

Please rate your use of the following international media platforms:

	Frequency of use						
**Social media**	Never	Several times a year	Once or twice a month	Once or twice a week	At least once a day	Several times a day	Almost every hour or more
1. Facebook	1	2	3	4	5	6	7
2. LinkedIn	1	2	3	4	5	6	7
3. WhatsApp	1	2	3	4	5	6	7
4. Twitter	1	2	3	4	5	6	7
5. Instagram	1	2	3	4	5	6	7
6. Tik Tok	1	2	3	4	5	6	7
7. Snapchat	1	2	3	4	5	6	7
8. Reddit	1	2	3	4	5	6	7
9. YouTube	1	2	3	4	5	6	7
10. Pinterest	1	2	3	4	5	6	7

Please, rate your use of the local social media platform/s (in your country):

Social media (Indicate what it is)	Never	Several times a year	Once or twice a month	Once or twice	At least once a day	Several times a day	Almost every hour or more

				a week			
1.	1	2	3	4	5	6	7
2.	1	2	3	4	5	6	7

What language do you use on social networks? To add list of frequent languages in Europe

How far do you use The Circle for:

Learning: a) I do not use it b) I use it for ______________________________

Networking with others: a) I do not use it b) I use it for _________________________

Thinking about your favorite two social media platforms please answer the following questions for each one (If you only use one platform, please complete the questions for this choice):

Learning

As a Sigma member, how far do you use your first choice for learning purposes* (including any kind of information, data or knowledge acquisition, which is not just of organizational nature)

Please answer using the following scale: 1 - Strongly Disagree, 2 - Disagree, 3 - Neither Agree nor Disagree, 4 - Agree, 5 - Strongly Agree

1. I use Social media to contact other members for learning purposes
2. I use Social media to post knowledge contents
3. I use Social media to receive knowledge contents (e.g. statements, links, materials)
4. I use Social media to discuss study related knowledge contents (actively)
5. I use Social media to follow discussions about knowledge contents (passively)
6. I would appreciate it if Sigma Europe/my Chapter used Social media as a teaching aid or tool
7. I do not use Social media (or at least not at all for learning purposes)

Free text

How else do you maybe use Social media for study related purposes?

Networking

As a Sigma member, how far do you use your first choice for networking purposes* (including interaction with others to exchange information and develop professional or social contacts)

Please answer using the following scale: 1 - Strongly Disagree, 2 - Disagree, 3 - Neither Agree nor Disagree, 4 - Agree, 5 - Strongly Agree

1. I use Social media to contact my peers for networking purposes
2. I use Social media to post information (e.g. about events, courses, conferences)
3. I use Social media to receive information (e.g. about events, courses, conferences)
4. I use Social media to promote myself professionally (actively)
5. I use Social media to follow others professionally (passively)
6. I would appreciate it if Sigma Europe/my Chapter used Social media as a networking tool
7. I do not use Social media (or at least not at all for networking purposes)

Free text

How else do you maybe use Social media to exchange information?

Part C

I would appreciate it if Sigma Europe offered other ways to share information

Yes/No

If yes, please state what ways you would like to see for

- Learning
- Networking

Part D

What are the main barriers for you in using social networks for learning or networking purposes?

____

____

____

____

The questionnaire was developed based on the combination of the following sources:

*Rigamonti L, Dolci A, Galetta F, Stefanelli C, Hughes M, Bartsch M, et al. Social media and e-learning use among European exercise science students. Health Promot Int. 2020;35(3):470–7. Available from: https://academic.oup.com/heapro/article/35/3/470/5487286

** Alharbi M, Kuhn L, Morphet J. Nursing students' engagement with social media as an extracurricular activity: An integrative review. J Clin Nurs. 2021;30(1–2):44–55. Available from: https://onlinelibrary.wiley.com/doi/10.1111/jocn.15503

**Alharbi M, Kuhn L, Morphet J. The relationship between social media usage by undergraduate nursing students and development of their professional identity: A correlational study. Nurse Educ Today. 2022;112:105337. Available from: https://linkinghub.elsevier.com/retrieve/pii/S0260691722000739

**Cathala X, Ocho ON, Watts PN, Moorley C. International student nurses' use of social media for learning: A cross sectional survey. Nurse Educ Today. 2021;107:105160. Available from: https://linkinghub.elsevier.com/retrieve/pii/S0260691721004172
