# Supplementary material for: Exploration of Social Media Usage Among Nurses in the European Region: A Cross-Sectional Study
Source: Comput Inform Nurs. 2026 Aug 20;44(11):e01616. doi: 10.1097/CIN.0000000000001616 (PMC13727034; doi:10.1097/CIN.0000000000001616)
Supplement: Supplementary file 3 [file nxn-44-e01616-s003.pdf]

Supplemental Table 1

The second popular social media platform (open-ended question*)

Social media platform	Frequency	Percent
**Facebook**	14	8,9
**None**	12	7,6
**Instagram**	11	7,0
Twitter	9	5,7
Linkedin	5	3,2
WhatsApp	5	3,2
I don't know*	3	1,9
Viber	2	1,3
The following were mentioned as free text once each: Copains d'Avant, Imessage, Local IG influencers, Meet, Quora, SharePoint*, Sigma chapter fb, Snapchat, TeamApp, Telegram, Threema, Tiktok, Viva Engage, You tube.	15	9,5
There're no local social media platformes used or unsure.	2	1,3
Missing	80	50,6
Total	158	100,0

* Not SM
