# Supplementary material for: Exploration of Social Media Usage Among Nurses in the European Region: A Cross-Sectional Study
Source: Comput Inform Nurs. 2026 Aug 20;44(11):e01616. doi: 10.1097/CIN.0000000000001616 (PMC13727034; doi:10.1097/CIN.0000000000001616)
Supplement: Supplementary file 4 [file nxn-44-e01616-s004.pdf]

Supplemental Table 2

How do the respondents use social media for learning (open-ended question)

Category	Use for	Frequency
To get information	R1 Sometimes being informed - forwarding all the information for our chapter	8
	R13 Information	
	R14 Information, research, networking	
	R27 Occasionally reading information posted by others	
	R28 one a week to keep abreast of information	
	R29 only for new information in the nursing category	
	R9 evidence-based scientific knowledge	
	R30 Prepare lessons	
To communicate and consult	R6 Contact network	6
	R32 Reach other members	
	R37 to communicate with other members	
	R7 Discussions, and questions from other members	
	R5 Consultation, carry out training	
	R33 Reading about the work of other nurses	
To access education, webinars and journals	R8 Educational learning	10
	R19 Learning	
	R20 Learning about new research projects	
	R21 learning on a global platform	
	R41 Watched training videos	
	R42 Webinars	
	R43 Webinars, CPD activities	
	R10 For information and participation in events	
	R2 Access to journal and cpd	
	R3 Access to webinars etc	

To develop or get ideas	R11 Get new ideas.	3
	R25 New developments in nursing	
	R24 Developing# ideas for healthcare	
To get updates	R12 Get updates	11
	R16 Keep up to date with some information about Sigma	
	R17 Keeping up to date - checking in with news etc	
	R18 Knowledge Update	
	R32 Updates on research	
	R35 Scientific updates	
	R34 Review current research	
	R38 Update	
	R39 Updates Interesting news webinars/masterclasses	
	R40 Updating on news	
	R36 Search for articles	
To network	R22 Network and learning	3
	R23 Networking and publicity of publications	
	R24 Networking.	
No or little	R26 No	4
	R31 Rarely	
	R15 Just joined	
	R4 Am new, not familiar yet	
Missing	-	115
	Total	n=158
