# Supplementary material for: Exploration of Social Media Usage Among Nurses in the European Region: A Cross-Sectional Study
Source: Comput Inform Nurs. 2026 Aug 20;44(11):e01616. doi: 10.1097/CIN.0000000000001616 (PMC13727034; doi:10.1097/CIN.0000000000001616)
Supplement: Supplementary file 5 [file nxn-44-e01616-s005.pdf]

Supplemental Table 3

Means for enhancing learning by Sigma

Category	Explanation	Frequency of responses per categoryn
1) Dissemination info about Sigma through websites, newsletters and meetings	R2 1°Greater dissemination of the SIGMA platform in health infrastructures<br>R3 A site for sharing knowledge from all over the world<br>R6 collaborative platforms other than sigma<br>R8 Designated web page with information links<br>R8 ...and maybe a monthly newsletter shared via social media with brief updates on activities and links to further info.<br>R3 Annual meeting<br>R14 Formation	7
2) Disseminating articles, facilitating access to articles	R20 relevant articles<br>R21 Research Gate<br>R22 Sharing articles and expertise.<br>R1 Links to articles and research<br>R2 2°Free access to literature reviews, primary and/or secondary nursing studies from around the world.	5

3) Ted-Talks, Podcasts, Webinars, Workshops, Digital Learning and policy skills	R24 Ted Talks style of lectures<br>R15 Hosting or advertising podcasts or Ted talks<br>R17 Lunch time talks available for all members<br>R18 Please make The Circle easier to use and navigate<br>R19 Podcast and webinar series<br>R29 Webinars<br>R7 Continuous training.<br>R31 Workshops, or short courses<br>R9 Digital learning for nurses<br>R12 Enhancing nursing skills and how to change policies!	10
4) Using diverse platform channels	R28 Use Facebook, Instagram, LinkedIn or tweet more, instead of circle<br>R27 Twitter content that is shareable<br>R23 Teams<br>R10 email<br>R16 I am happy for the e-mails with information form Sigma Europe and my Tau chapter and for the web-sites where I can sign in at Sigma<br>R11 email youtube<br>R13 facebook, instagram<br>R5 At least through Instagram and Facebook.<br>R30 What's app	11

	R32 YouTube	
	R33 Zoom meetings	
Missing	n= 121 comments	
**Total**	**n= 158; 100%**	**33**
