# Supplementary material for: Exploration of Social Media Usage Among Nurses in the European Region: A Cross-Sectional Study
Source: Comput Inform Nurs. 2026 Aug 20;44(11):e01616. doi: 10.1097/CIN.0000000000001616 (PMC13727034; doi:10.1097/CIN.0000000000001616)
Supplement: Supplementary file 6 [file nxn-44-e01616-s006.pdf]

Supplemental Table 4

Barriers and challenges for networking by using social media

**Category**	**Explanation of the barrier**	**Frequency of phrases per category**
1. Lack of time	R8 don't have time<br>R78 time<br>R59 I do not have enough time to follow them all.<br>R22 I don't have enough time<br>R24 I don't use it for learning<br>R30 It is difficult to find time for it.<br>R31 It takes up to much time<br>R36 Lack of time (two respondents)<br>R41 lack of time to engage<br>R42 Lack of time to use social media.<br>R43 Lack of time.<br>R50 Not enough time for social media use.<br>R37 Lack of time -> Not enough time to search for information as much as I would like to. I am not familiar with all available social network platforms.<br>R38 lack of time and too complex.<br>R70 time<br>R67 The time it takes using the phone.<br>R71 Time<br>R79 Time! I do not have the time for being on social medias.<br>R80 Timing<br>R72 Time, I sufficient tech support.<br>R73 Time and nervousness of social media usage<br>R74 Time challenges<br>R75 Time consuming<br>R76 Time, changes to algorithms such as Twitter since Elon Musk takeover<br>R77 Time,	30

	R40 Lack of time to balance work and family life with all the channels through which information and requests constantly reach us.<br>R87 time<br>R88 time<br>R56 right now I have a lot of obligations, and I can't make it	
2. No barriers	R21 I can't really think of many<br>R23 I don't identify barriers.<br>R26 I have no barriers<br>R47 No<br>R48 None (7 respondents)<br>R1 None<br>R46 Networking<br>R49 None really<br>R68 There are no barriers, as long as private messages are part of the services<br>R94 I see no need.	16
3. Lacking knowledge or fear of lacking skills to use	R11 Fear<br>R77 Fear of negative comments<br>R12 Fear of using it Lack of 'know how'<br>R32 Knowledge of how to use them effectively<br>R33 knowledge, experience and time<br>R35 Lack of social media and computer knowledge and confidence. Time is also a challenge<br>R89 Awareness of it happening<br>R20 I am retired so I have limited access and limited skills using social media and have a bit reduced interest in learning and networking purposes but like to be informed about whats happening in the nursing profession, though! So thank you to Sigma Europe!<br>R85 understanding new technologies<br>R86 using several different platforms makes it more difficult to navigate, therefore, I recently quit Twitter and focus on LinkedIn	10

	use for professional purposes and facebook for private networking purposes only	
4. Poor security and privacy uncertainty	R39 Security also an issue<br>R52 Privacy (two respondents)<br>R25 I dont want to use my private social network for work related purposes<br>R16 GDPR /Privacy / Consent<br>R53 Privacy concerns. I prefer privacy-oriented platforms whenever possible, eg. Signal.<br>R54 Public sharing is not safe enough<br>R55 Quality assurance of the information being provided.<br>R57 Security	9
5. Too much information and channels	R15 Frequent or unnecessary interruptions from adds or posts<br>R51 Overload of information and too frequently<br>R82Too many adverts on social media<br>R83 Too much information and too much time spent on social media are problems that I am dealing with.<br>R84 Too much information overload as too busy to keep up with everything. I miss stuff that comes through when busy even if I would like to read it.<br>R69 There are so many platforms, so much information. It's hard to keep up.<br>R19 I am fully occupied communicating with colleagues using the Circle, and email and find it too demanding to have so many different forms of communicating with colleagues.<br>R59 Sometimes it feels like there is too many social media channels to use<br>R63 The existence of several networks and consequent dispersion	9
6. Connectivity, costs of data, accessibility, Format not interactive	Response 5 [R5]* Connectivity<br>R29 Internet connectivity and cost of data<br>R7 Difficulty accessing pertinent information<br>R9 Ease of access. Some feel very 'American-centric' which is not inclusive or encouraging.<br>R79 needs to be easily accessed and non intrusive	7

	R18 Homepage of difficult access<br>R64 The existing format does not favor interaction.	
7. Lack of quality, reliability, credibility and content	R4 Check if sources are reliable<br>R6 credible sources<br>R13 Filtering the information available to determine relevant content<br>R34 Lack of credibility in information<br>R65 The lack of content.	5
8. Opportunistic posting, unrelated or poor content, oversharing personal needs, wants and opinions, marketing	R81 To many non-specific content<br>R66 The lack of quality/veracity of the clinical information that is shared.<br>R60 Sometimes its use is not consensual, nor is it recommended by institutions.<br>R62 The existence of opportunistic people who use groups/networks to publish commercial matters or content unrelated to the group's objective<br>R78 poorly informed posts, I get irritated with people who overshare there personal needs, wants and opinions, I particularly dislike media warriers	5
9. Languages	R10 English language<br>R44 language<br>R45 Language<br>R58 Sometime language	4
**Missing**	**n=58**	58
**Removed irrelevant responses**	R90, R91, R92, R93, R95 (not describing barriers)	5
**Total**	**n=158**	**158**

*Number of the response (R) stands for the consecutive number of responses to the research question; thus, it does not stand for the responding individual.
